# Increased lipogenesis and lipidosis of gallbladder epithelium in dogs with gallbladder mucocele formation

**DOI:** 10.1371/journal.pone.0303191

**Published:** 2024-06-26

**Authors:** Jody L. Gookin, Dennis E. Jewell, Kathleen M. Aicher, Gabriela S. Seiler, John M. Cullen, Kyle G. Mathews

**Affiliations:** 1 Department of Clinical Sciences, College of Veterinary Medicine, North Carolina State University, Raleigh, NC, United States of America; 2 Department of Grain Science and Industry, Kansas State University, Manhattan, KS, United States of America; 3 Department of Small Animal Clinical Sciences, College of Veterinary Medicine, Texas A & M University, College Station, TX, United States of America; 4 Department of Molecular Biomedical Sciences, College of Veterinary Medicine, North Carolina State University, Raleigh, NC, United States of America; 5 Department of Population Health and Pathobiology, College of Veterinary Medicine, North Carolina State University, Raleigh, NC, United States of America; Ann and Robert H Lurie Children’s Hospital of Chicago / Northwestern University Feinberg School of Medicine, UNITED STATES

## Abstract

**Background:**

Gallbladder disease in people is frequently associated with disorders of lipid metabolism and metabolic syndrome. A recently emergent gallbladder disease of dogs, referred to as mucocele formation, is characterized by secretion of abnormal mucus by the gallbladder epithelium and is similarly associated with hyperlipidemia, endocrinopathy, and metabolic dysfunction. The cause of gallbladder mucocele formation in dogs is unknown.

**Methods:**

A prospective case-controlled study was conducted to gain insight into disease pathogenesis by characterization of plasma lipid abnormalities in 18 dogs with gallbladder mucocele formation and 18 age and breed matched control dogs using direct infusion mass spectrometry for complex plasma lipid analysis. This analysis was complemented by histochemical and ultrastructural examination of gallbladder mucosa from dogs with gallbladder mucocele formation and control dogs for evidence of altered lipid homeostasis of the gallbladder epithelium.

**Results:**

Gallbladder mucocele formation in dogs carried a unique lipidomic signature of increased lipogenesis impacting 50% of lipid classes, 36% of esterified fatty acid species, and 11% of complex lipid species. Broad enrichment of complex lipids with palmitoleic acid (16:1) and decreased abundance within complex lipids of presumptive omega-3 fatty acids eicosapentaenoic (20:5) and docosahexaenoic (22:6) was significant. Severe lipidosis of gallbladder epithelium pinpoints the gallbladder as involved causally or consequently in abnormal lipid metabolism.

**Conclusion:**

Our study supports a primary increase in lipogenesis in dogs with mucocele formation and abnormal gallbladder lipid metabolism in disease pathogenesis.

## Introduction

Gallbladder mucocele formation is a disease unique to the dog that is characterized by mucosal cystic hypertrophy and increased secretion of gel-forming mucin by the gallbladder epithelium [1]. Over time, amassment of abnormally thick mucus is associated with impaired gallbladder motility, extrahepatic biliary tract obstruction, and gallbladder rupture. For affected dogs, surgery to remove the gallbladder can be lifesaving. However, an average of 20% of dogs will die or be euthanized within 2 weeks of hospitalization due to post-operative complications [2–4].

The cause of gallbladder mucocele formation in dogs is unknown. An interesting observation in dogs that develop gallbladder mucocele formation is an increased likelihood for concurrent diagnosis of hyperlipidemia, endocrinopathy, and metabolic disruption[2, 5–11]. Hypercholesterolemia or hypertriglyceridemia is identified in 90% of dogs with gallbladder mucocele formation[2]. We surmised that further insight into gallbladder mucocele pathogenesis might be gained by characterization of the disturbance in lipid metabolism observed in these dogs. Yet, description of hyperlipidemia in dogs with gallbladder mucocele formation is confounded by the concurrent influence of hyperadrenocorticism, hypothyroidism, and cholestasis on lipid metabolism in up to 25% of these dogs [2, 5, 10].

Accordingly, the objective of this case-controlled study was to prospectively characterize plasma lipid abnormalities in dogs with gallbladder mucocele formation in which the influence of concurrent endocrinopathy deemed unlikely by comprehensive thyroid and adrenal function testing. Our approach utilized direct infusion mass spectrometry for complex plasma lipid analysis. These studies were complemented by histochemical and transmission electron microscopic examination of gallbladder mucosa from dogs with gallbladder mucocele formation and control dogs for evidence of altered lipid homeostasis of the gallbladder epithelium.

## Materials and methods

### Prospective case and control selection criteria

Dogs meeting ultrasonographic criteria [12] for diagnosis of gallbladder mucocele formation were prospectively identified from February 2014 to January 2017. An apparently healthy, age, and breed-matched cohort of client-owned dogs were concurrently recruited for inclusion as controls. For each control dog, ultrasonography was used to confirm absence of gallbladder mucocele formation based on a normal appearing gallbladder with normal wall structure and thickness. Sludge, if present, was gravity-dependent, occupied less than 50% of the gallbladder lumen, and was not attached to the wall.

Medical records were reviewed, and dogs were excluded if they had a prior diagnosis or were currently receiving treatment for hypothyroidism, hyperadrenocorticism, or diabetes mellitus. Each dog additionally underwent thyroid and adrenal cortical function testing by means of comprehensive serum thyroid hormone profiling (Michigan State University Veterinary Diagnostic Laboratory, Endocrinology Section, Lansing, MI) and measurement of serum cortisol concentration 1 hour after intravenous injection of synthetic cosyntropin (Cortrosyn®, Amphastar Pharmaceuticals, Inc, Rancho Cucamonga, CA) as previously described [10]. Dogs were excluded if they met criteria for diagnosis of hypothyroidism or hyperadrenocorticism as previously described [5, 13, 14]. Dogs were additionally excluded if they had a recent (within 2 months) history of treatment with ursodeoxycholic acid or drugs recognized or suspected to interfere with thyroid or adrenal function testing (e.g., topical or systemic glucocorticoids, anti-convulsant, furosemide, sulfa-containing drugs, fatty acid supplements) or were reproductively intact. Reproductively intact dogs were excluded from the study to eliminate any confounding influence on lipid metabolism. Dogs were not matched by sex.

All dogs underwent a complete physical examination by the attending clinician. Each dog was stratified by disease severity into four groups based on a previously described scoring system [15] as follows: absent (0) for patients that demonstrated no clinical signs of illness, mild (1) for patients with signs of clinical disease but suitable for outpatient care, moderate (2) for patients sick enough to require hospitalization and aggressive treatment, and severe (3) for patients with severe illness requiring intensive care and advanced treatment. Blood was collected by means of venipuncture after a minimum fasting period of 12 hours. Anticoagulated (EDTA) whole blood, plasma, and serum were processed by the NCSU-VH Clinical Pathology Laboratory for a complete blood cell count and serum biochemical analysis. Aliquots of plasma for lipidomics analysis were stored at -80°C within 30-min of collection and archived for approximately 2 years prior to analysis.

Owners of each dog signed a written informed consent for participation in the study. All study protocols were approved by the Institutional Animal Care and Use Committee of North Carolina State University (ID#14-049-O).

### Infusion mass spectrometric analysis of plasma complex lipids

Complex lipids analysis was performed by a commercial laboratory (Metabolon Inc. Morrisville NC 27560). Lipids were extracted in the presence of deuterated internal standards using an automated BUME extraction according to the method of Lofgren et al. [16]. The extracts were concentrated under nitrogen and reconstituted in 0.25 mL of 10 mM ammonium acetate dichloromethane:methanol (50:50). The extracts were transferred to inserts and placed in vials for infusion-MS analysis, performed on a Shimazdu LC with nano PEEK tubing and the Sciex SelexIon-5500 QTRAP. The samples were analyzed via both positive and negative mode electrospray. The 5500 QTRAP scan was performed in MRM mode with a total of more than 1,100 MRMs. Individual lipid species were quantified by taking the peak area ratios of target compounds and their assigned internal standards, then multiplying by the concentration of internal standard added to the sample. Lipid class concentrations were calculated from the sum of all molecular species within a class, and fatty acid compositions were determined by calculating the proportion of each class comprised of individual fatty acids.

### Light and transmission electron microscopy

Full-thickness sections of gallbladder mucosa were embedded in optimal cutting temperature medium, frozen in liquid nitrogen and sectioned at a thickness of 10 μm prior to mounting on glass slides. Sections were dried overnight, fixed in 10% neutral buffered formalin and rinsed prior to treatment with 30% and 60% isopropanol, a 15-min staining with Oil-Red-O, treatment with 60% and 30% isopropanol, rinse and counterstain with Mayer’s hematoxylin.

For transmission electron microscopy, samples were rinsed in 0.1M sodium phosphate buffer (pH 7.2) and placed in 1% osmium tetroxide in the same buffer for 1 hour at room temperature. Samples were rinsed two times in distilled water and were dehydrated in an ethanol series culminating with two changes of 100% acetone. Tissues were then placed in a mixture of Spurr resin and acetone (1:1) for 30 minutes, followed by 2 hours in 100% resin with two changes. Finally, tissues were placed in fresh 100% resin in molds and were polymerized at 70°C for 8 hours to 3 days. Semithin (0.25–0.5 mm) sections were cut with glass knives and stained with 1% toluidine blue-O in 1% sodium borate. Ultrathin (70–90 nm) sections were cut with a diamond knife, stained with methanolic uranyl acetate followed by lead citrate, and examined using a FEI/Philips EM 208S transmission electron microscope.

### Data analysis

Data were examined using a Shapiro-Wilk test for normality to determine choice for use of parametric versus non-parametric statistical testing. Complete blood cell counts and serum biochemistry findings were summarized as median and range and tested for significant differences between control and gallbladder mucocele dogs using a Mann-Whitney Rank Sum test. Differences in percentages of dogs with abnormal values were tested using a Chi-square or Fisher Exact test with p<0.05 considered significant. Lipid class and fatty acid concentrations and mol % composition data were summarized as median and interquartile range. Differences between control dogs and dogs with gallbladder mucocele formation were examined for statistically significant differences (p <0.05) using a Mann-Whitney Rank Sum test (SigmaPlot Version 14), followed by application of a Benjamini-Hochberg correction [17] for false discovery at p<0.15.

Global statistical analysis of the 981 unique complexes of class and esterified fatty acid(s) lipid species measured was conducted via the Metabolon Surveyor platform in which fold change differences between control and gallbladder mucocele groups was calculated and a Welch’s two-sample *t*-test was applied. This version of the two-sample *t*-test allows for unequal variances and has a *t*-distribution with degrees of freedom estimated using Satterthwaite’s approximation. Statistical significance was assessed at a liberal uncorrected p-value <0.05. Measurements obtained from individual dogs were graphed using GraphPad Prism 9.

## Results

### Clinical findings

Fasting plasma samples were prospectively collected from 18 dogs with gallbladder mucocele formation and 18 age and breed-matched control dogs. Concurrent hypothyroidism or hyperadrenocorticism was determined to be unlikely for each dog based on normal results obtained from comprehensive thyroid hormone profile and cosyntropin stimulation testing. Dogs with gallbladder mucocele formation ranged from 6 to 15 years in age (median, 9.5 years) and were represented by 12 breeds including Shetland Sheepdog (7 dogs), American Cocker Spaniel, American Staffordshire Terrier, Bichon Frise, Border Collie, Cavalier King Charles Spaniel, Chihuahua, Cockapoo, Fox Terrier, Labrador Retriever, Miniature Schnauzer, and Pug. Six dogs were spayed females and 12 were neutered males. Illness severity scores of the dogs at the time of participation in the study were as follows: 0 (absent) in 10 dogs, 1 (mild) in 2 dogs, 2 (moderate) in 4 dogs, and 3 (severe) in 2 dogs.

**Fig 1:**
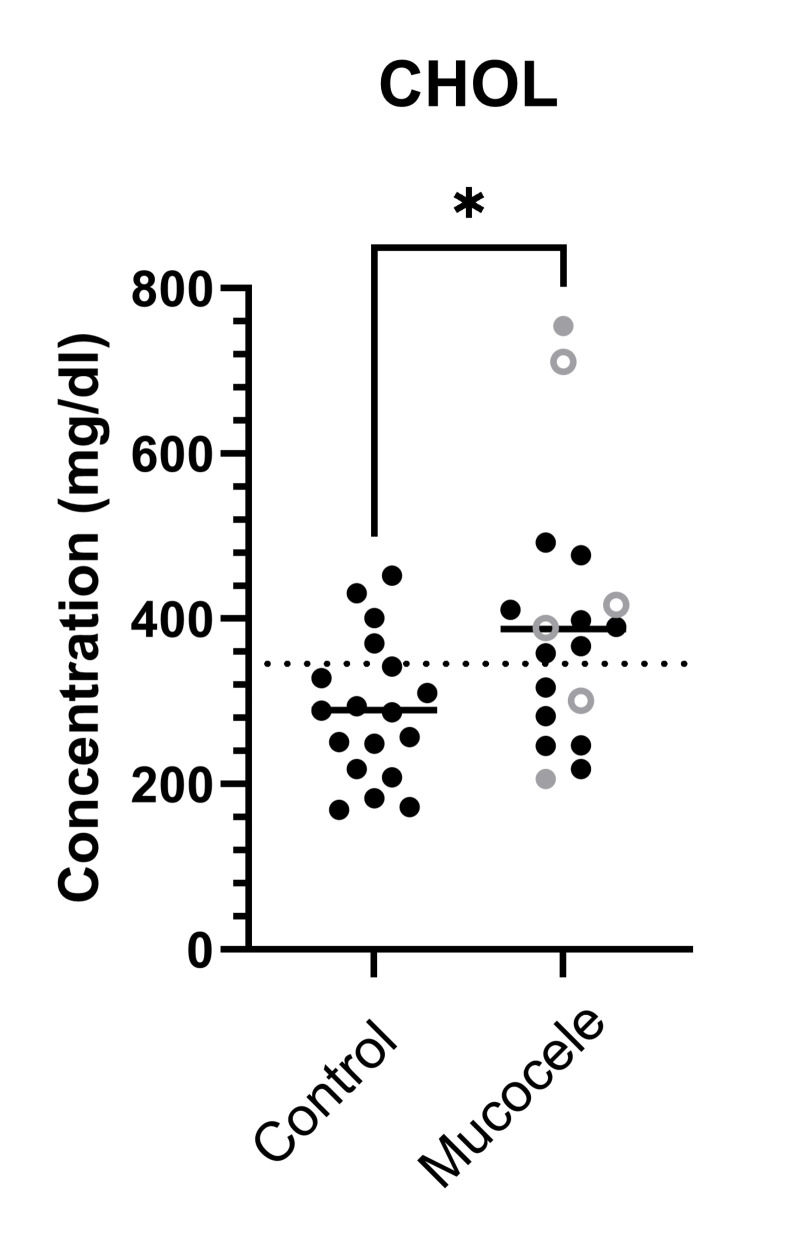
Fasting serum cholesterol (CHOL) concentrations in 18 dogs with gallbladder mucocele formation and 18 dogs matched by age and breed. Open circles represent dogs having serum biochemical evidence of cholestasis as defined by a serum total bilirubin concentration greater than the upper end of the reference range (>0.2 mg/dl). Gray datapoints represent dogs with illness severity score ≥ 2. Mann-Whitney P value *<0.05.

Control dogs ranged from 6 to 13 years in age (median, 10 years). Seven dogs were spayed females and 11 were neutered males. A summary of diet and medication history obtained from medical records of dogs in the study is shown in **S1 Table**. Compared to control dogs, results of complete blood cell count and serum biochemistry analysis in dogs with gallbladder mucocele formation demonstrated significant increases in number of polymorphonuclear leukocytes, and concentrations of liver and pancreatic enzyme activities, total bilirubin, and cholesterol. Hypercholesterolemia was documented in 61% of dogs with gallbladder mucocele formation (**Table 1** and **Fig 1**).

**Table 1:** Selected complete blood cell count and serum biochemical analysis findings in 18^§^ dogs with gallbladder mucocele formation and 18 control dogs that fit inclusion criteria for this study.

Clinical Pathological Variable	No Gallbladder Mucocele (18 dogs)			Gallbladder Mucocele (18 dogs^§^)			Reference range
	Median	Range	Number (%) of dogs with abnormal value	Median	Range	Number (%) of dogs with abnormal value	
**Complete blood cell count**							
Packed cell volume (%)	43	30–51	11.1	44	25–56	12.5	39–58
Plasma protein (g/dl)	7.2	5.8–8.3	44.4	7.9	6.3–10	61.5	6.1–7.5
Total white blood cells (× 10^3^/μl)	7.015	4.490–12.590	11.1	8.68	4.67–34.570	29.4	4.39–11.61
Polymorphonuclear leukocytes (PMN) (× 10^3^/μl)	5.005	2.371–10.450	5.6	**6.705** *	3.468–28.926	29.4	2.841–9.112
Immature PMN (× 10^3^/μl)	0	0–210	33.3	**204** *	0–6.568	64.7	0.0–0.0
Platelets (× 10^3^/μl)	315.5	253–383	5.6	384	143–564	11.8	191–468
**Serum biochemical analysis**							
Alkaline phosphatase (IU/L)	51	6–213	11.1	**199** **	21–3188	**66.7** **	16–140
ALT (IU/L)	48	11–88	27.8	**118** *	16–5393	**66.7** *	12–54
GGT (IU/L)	0	0–4	0	**5.5** **	0–112	**44.4** **	0–6
Total bilirubin (mg/dl)	0	0–0.1	0	**0.1** ***	0–11.5	22.2	0–0.2
Cholesterol (mg/dl)	288	169–452	22.2	**378** *	206–754	**61.1** *	124–344
Blood urea nitrogen (mg/dl)	15	8–36	5.6	15	5–170	11.1	8–26
Creatinine (mg/dl)	0.7	0.5–1.1	0	0.7	0.2–4.1	5.6	0.7–1.5
Albumin (g/dl)	3.6	2.9–4.2	5.6	3.4	2.1–4.2	16.7	3–3.9
Lipase (IU/L)	58	24–633	5.6	**119** *	41–1558	**38.9** *	12–147
Amylase (IU/L)	633	67–1496	5.6	**804** *	338–2054	22.2	236–1337

Comparison of median values performed using Mann-Whitney Rank Sum Test. Comparison of proportions performed using Chi-square or Fisher Exact statistic.

*p<0.05

**p<0.01, and

***p<0.001.

^§^ Complete blood cell count was not performed in 1 dog with gallbladder mucocele formation

PMN, polymorphonuclear leukocytes; ALT, alanine aminotransferase; GGT, gamma-glutamyl transferase

### Plasma complex lipids

A total of 14 classes of neutral lipids, phospholipids, and sphingolipids, 28 different saturated, monounsaturated, and polyunsaturated esterified fatty acids, and 981 unique species of class and esterified fatty acid(s) combinations of lipids were measured in plasma from dogs in this study (**S2 Table**).

### Lipid classes

**Fig 2:**
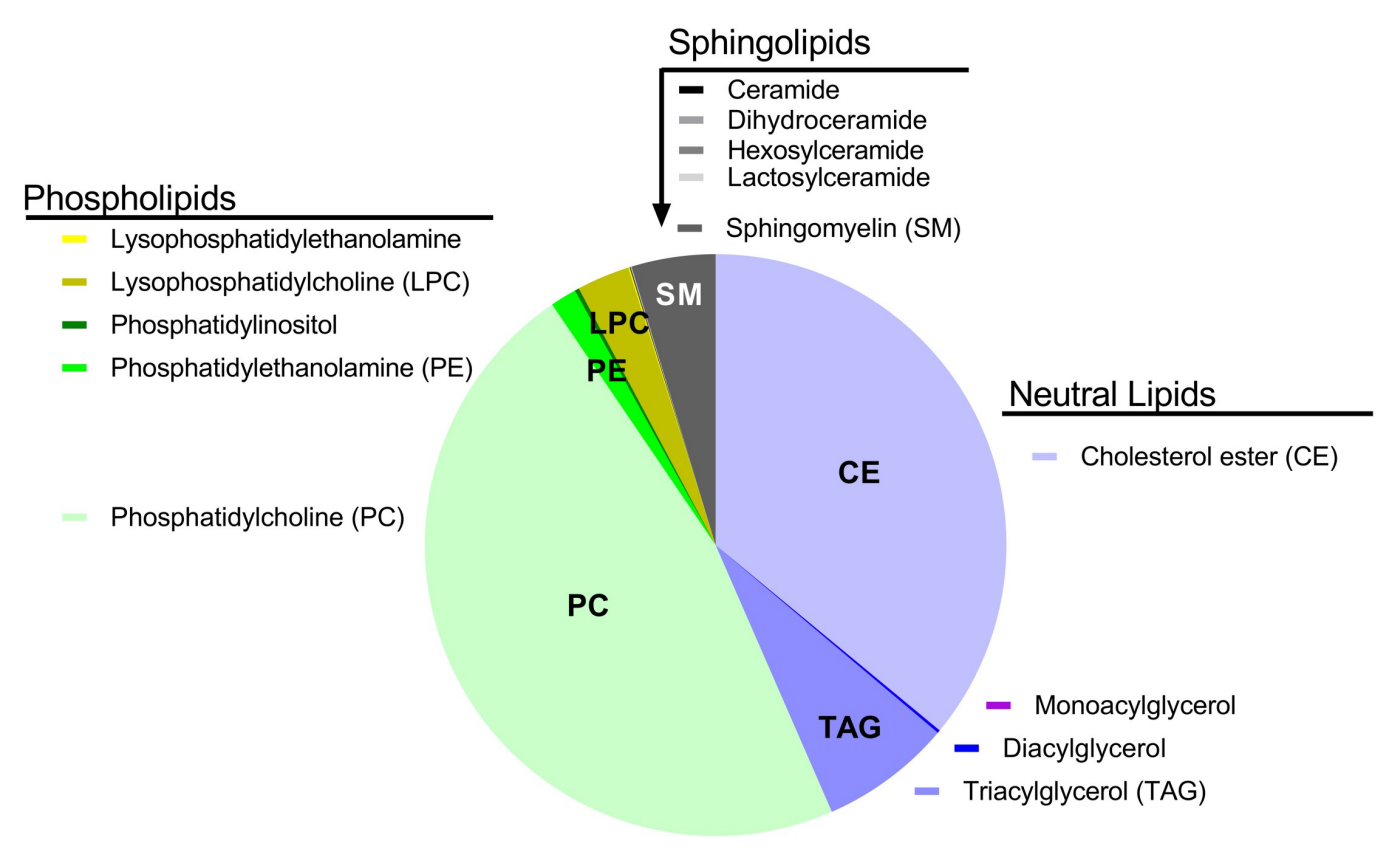
Percent abundance of classes of complex lipids identified in fasted plasma samples from dogs in this study.

Significant increases were measured in class concentrations (nmol/ml) of cholesterol ester, monoacylglycerol, phosphatidylcholine, lysophosphatidylethanolamine, dihyroceramide, hexosylceramide, and sphingomyelin in plasma from dogs with gallbladder mucocele formation (**Table 2**). Significant differences in triacylglycerol concentrations in plasma from dogs with gallbladder mucocele formation was not observed (S1 Fig). Relative abundance (mol %) of lipid classes was not significantly different between control dogs and dogs with gallbladder mucocele formation and was largely represented by phosphatidylcholine, cholesterol esters, triacylglycerides and sphingomyelin (**Fig 2** and **Table 2**).

**Table 2:** Lipid class concentrations and relative abundances measured in the plasma of 18 control dogs and 18 dogs with gallbladder mucocele formation.

Lipid Class		Lipid Class Concentration (nmol/ml)						Mann-Whitney Rank Sum p-value	Lipid Class Composition (mol % of total lipid classes)						Mann-Whitney Rank Sum p-value
		No Gallbladder Mucocele (n = 18)			Gallbladder Mucocele (n = 18)				No Gallbladder Mucocele (n = 18)			Gallbladder Mucocele (n = 18)			
		Median	IQR		Median	Median			Median	Median		Median	Median		
			Q1	Q3		Q1	Q3			Q1	Q3		Q1	Q3	
Neutral Lipids															
CE	Cholesterol ester	2979.4965	2332.2452	3400.4691	3877.5076	3002.7370	4154.9980	0.026*	35.7111	32.8859	37.7296	35.4933	32.3008	35.8138	0.420
MAG	Monoacylglycerol	1.2583	1.0538	1.6335	1.8500	1.4491	2.1970	0.020*	0.0165	0.0152	0.0180	0.0174	0.0153	0.0189	0.476
DAG	Diacylglycerol	10.5158	7.6661	16.5430	17.0061	13.3295	22.8479	0.060	0.1505	0.1076	0.1817	0.1447	0.1257	0.1981	0.716
TAG	Triacylglycerol	490.8992	358.8211	941.7173	876.7253	576.6434	1613.8171	0.150	7.2401	4.1557	10.7799	7.5131	5.1717	13.3036	0.420
Phospholipids															
PC	Phosphatidylcholine	3862.6447	2923.6842	4843.0118	5066.5423	4438.1049	5788.2280	0.005*	46.6773	44.4700	47.9238	47.1391	44.7015	49.4261	0.367
PE	Phosphatidylethanolamine	118.7616	93.9868	154.8414	156.8811	113.0388	206.8659	0.056	1.4526	1.3479	1.6527	1.3918	1.2372	1.7518	0.624
PI	Phosphatidylinositol	22.1117	14.5432	29.2220	26.1574	19.0001	33.6158	0.289	0.2403	0.2188	0.2881	0.2720	0.1970	0.2897	0.937
LPC	Lysophosphatidylcholine	245.6082	208.6102	303.6350	297.1260	229.6708	329.1565	0.275	2.9171	2.6437	3.3990	2.6724	2.5077	2.8746	0.024
LPE	Lysophosphatidylethanolamine	6.5865	4.9949	8.2466	8.8777	7.0784	10.4470	0.008*	0.0787	0.0751	0.0850	0.0834	0.0765	0.0899	0.206
Sphingolipids															
CER	Ceramide	3.6007	3.3442	3.7812	3.7415	2.9197	4.6284	0.351	0.0375	0.0339	0.0494	0.0346	0.0281	0.0421	0.125
DCER	Dihydroceramide	1.3089	1.1732	1.5562	1.6717	1.3971	1.8841	0.006*	0.0161	0.0143	0.0172	0.0149	0.0131	0.0161	0.282
HCER	Hexosylceramide	1.2005	1.0732	1.3095	1.5098	1.3028	1.8709	0.006*	0.0174	0.0112	0.0185	0.0143	0.0119	0.0176	0.692
LCER	Lactosylceramide	2.5677	2.1367	3.0634	2.9997	2.1815	4.0135	0.477	0.0349	0.0225	0.0430	0.0283	0.0189	0.0396	0.438
SM	Sphingomyelin	321.5615	296.9474	378.1881	403.6115	365.4472	478.9715	0.012*	4.6376	3.4907	4.9258	3.7029	3.2334	4.6398	0.200

*Retained significance at a Benjamini-Hochberg corrected p-value <0.15.

### Esterified fatty acids

**Fig 3:**
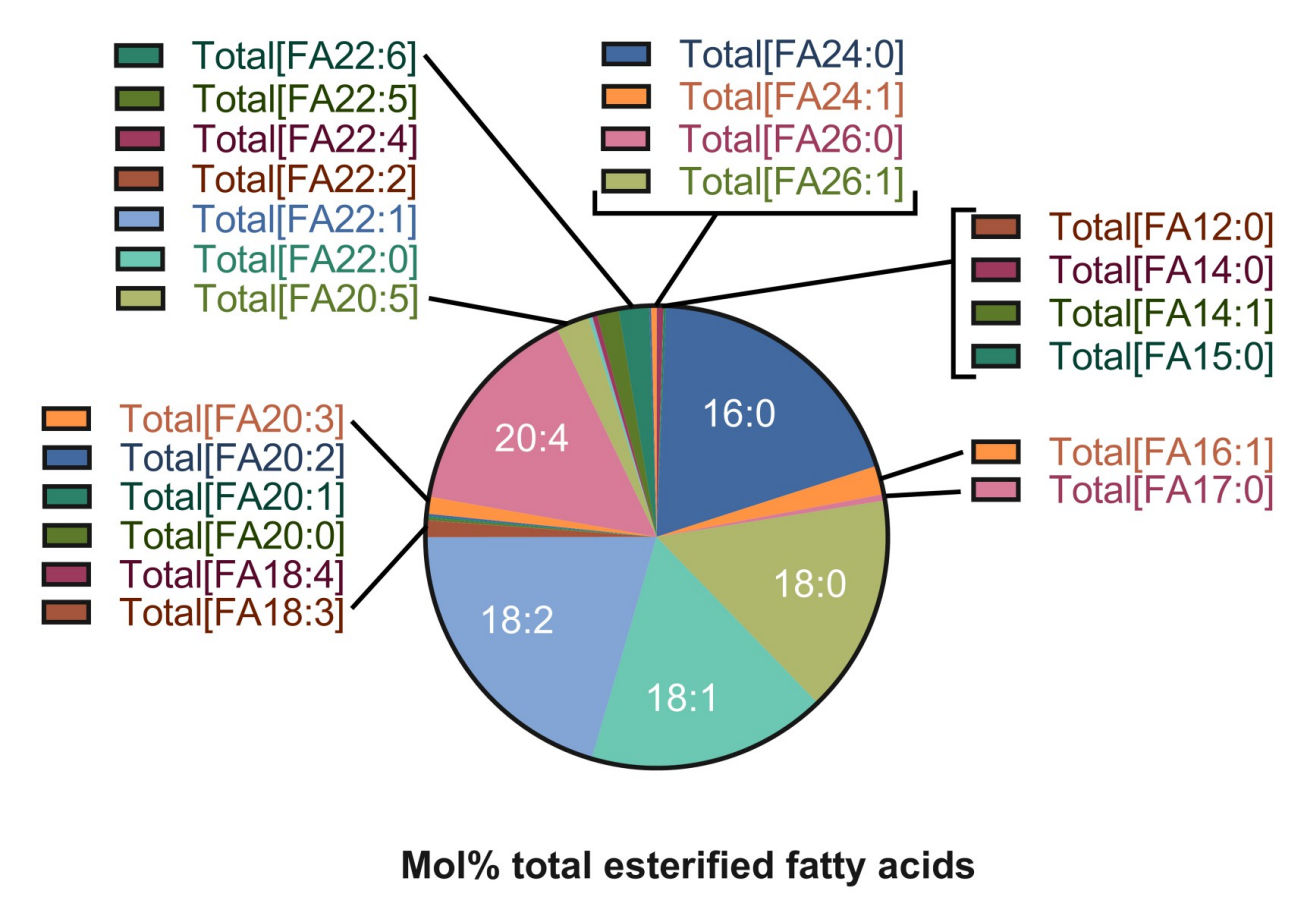
Percent total abundance of esterified fatty acid species identified in fasted plasma samples from 18 control dogs in this study.

**Fig 4:**
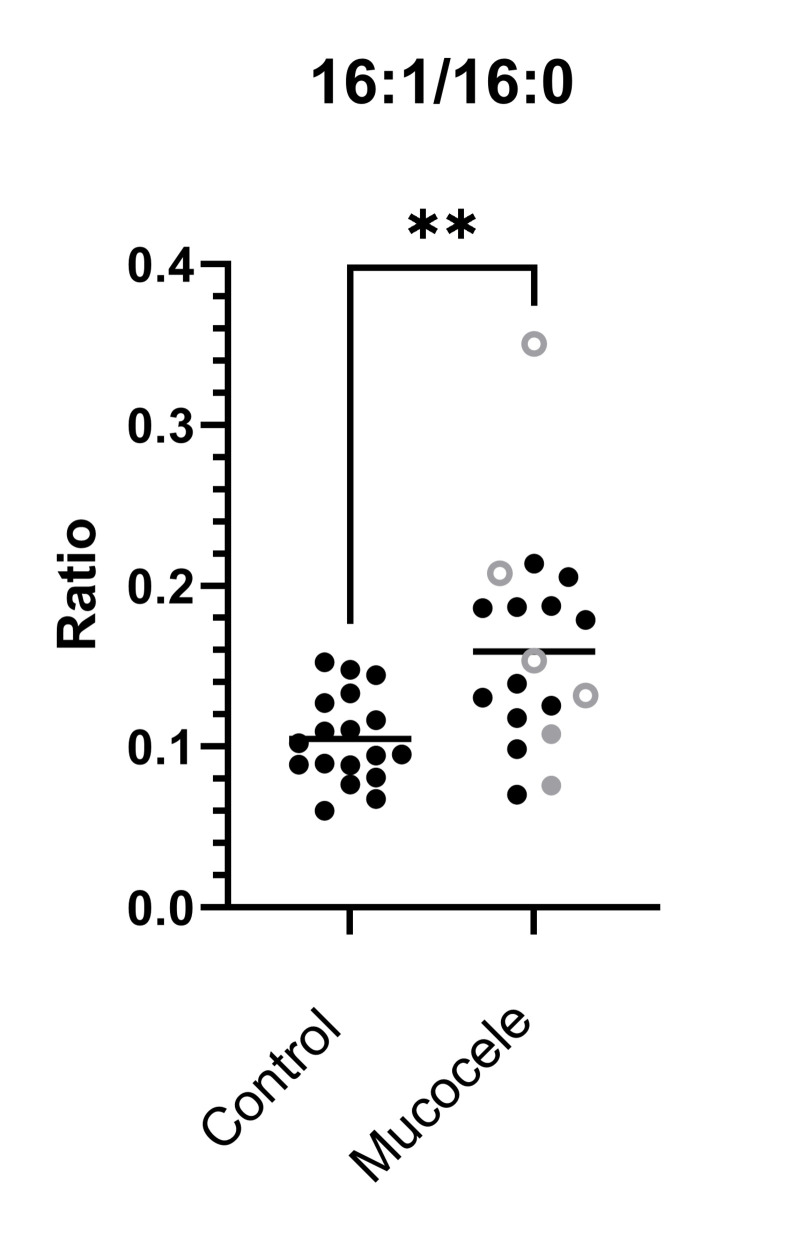
A significant increase in the ratio of palmitoleic acid to palmitic acid (16:1/16:0) in dogs with gallbladder mucocele formation compared to age and breed matched control dogs. Open circles represent dogs having serum biochemical evidence of cholestasis as defined by a serum total bilirubin concentration greater than the upper end of the reference range (>0.2 mg/dl). Gray datapoints represent dogs with illness severity score ≥ 2. Mann-Whitney P value **<0.01.

Twenty-eight different species of esterified fatty acids were identified in the plasma of dogs in this study (**Fig 3**). Significant increases were measured in the plasma concentration of 10/28 (36%) esterified fatty acid species in dogs with gallbladder mucocele formation. Among these fatty acids, palmitoleic (16:1) was additionally present at a significantly greater relative abundance (mol %) compared to other fatty acids and increased conversion of palmitic acid (16:0) to palmitoleic acid (16:1) was supported by an increased 16:1/16:0 ratio (**Table 3** and **Fig 4**). No fatty acid species was identified as significantly decreased in plasma concentration in dogs with gallbladder mucocele formation. However, two odd-chain fatty acids, pentadecanoic (15:0) and margaric (17:0), and the omega-3 fatty acid docosahexaenoic (22:6) were present at a significantly lower relative abundance (mol %) compared to other fatty acids (**Table 3**).

**Table 3:** Esterified fatty acid concentrations and relative abundance measured in the plasma of 18 control dogs and 18 dogs with gallbladder mucocele formation.

Fatty Acid Species		Fatty Acid Concentration (nmol/ml)						Mann-Whitney Rank Sum p-value	Fatty Acid Composition (mol % of total fatty acids)						Mann-Whitney Rank Sum p-value
		No Gallbladder Mucocele (n = 18)			Gallbladder Mucocele (n = 18)				No Gallbladder Mucocele (n = 18)			Gallbladder Mucocele (n = 18)			
		Median	IQR		Median	IQR			Median	IQR		Median	IQR		
			Q1	Q3		Q1	Q3			Q1	Q3		Q1	Q3	
**12:0**	Lauric	1.0621	0.7491	3.2650	1.2500	0.9719	2.4627	0.624	0.0084	0.0068	0.0196	0.0082	0.0061	0.0097	0.319
**14:0**	Myristic	45.5686	40.2143	94.2111	48.0024	39.0636	72.9154	0.962	0.4179	0.3342	0.5185	0.2959	0.2514	0.3997	0.028
**15:0**	Pentadecanoic	20.8095	15.0697	26.0631	18.5192	13.6487	29.5299	0.788	0.1575	0.1151	0.1965	0.1193	0.0740	0.1464	0.016*
**16:0**	Palmitic	2442.4501	2041.0467	3741.1533	3312.4834	2367.9253	4168.2534	0.200	19.1933	17.6574	21.3104	17.9193	16.0410	20.1090	0.091
**17:0**	Margaric	58.8676	42.2417	74.3318	50.9481	38.8312	96.9198	0.887	0.4161	0.3378	0.5184	0.3295	0.2682	0.3699	0.014*
**18:0**	Stearic	2121.2186	1757.9959	2677.5980	2895.3313	2629.5326	3465.3636	0.014*	14.9293	14.1497	16.6627	15.6455	14.7661	16.5738	0.457
**20:0**	Arachidic	22.9110	19.3229	25.7155	25.5462	21.6066	34.4100	0.189	0.1597	0.1367	0.1822	0.1479	0.1248	0.1577	0.064
**22:0**	Behenic	27.8554	21.6419	32.9682	42.5121	30.7745	47.4763	<0.001*	0.1943	0.1645	0.2255	0.2344	0.1781	0.2572	0.200
**24:0**	Lignoceric	16.7001	13.9281	19.7673	21.2946	18.8082	23.6595	0.008*	0.1358	0.1067	0.1569	0.1113	0.0906	0.1439	0.235
**26:0**	Hexacosanoic	0.4990	0.4287	0.5796	0.6086	0.5542	0.7531	0.005*	0.0038	0.0030	0.0043	0.0034	0.0026	0.0047	0.874
**14:1**	Myristoleic	3.5382	1.8681	7.6302	3.5491	2.6746	7.3509	0.457	0.0302	0.0166	0.0433	0.0232	0.0165	0.0345	0.681
**16:1**	Palmitoleic (n-7)	254.2010	179.5165	376.6453	391.3950	340.0552	619.6614	0.018*	1.8960	1.6835	2.2694	2.5711	2.0774	3.0242	0.009*
**18:1**	Octadecaenoic (Oleic)	2167.6881	1621.7242	2651.2640	3289.2490	2569.1935	4293.2416	0.022*	16.5216	15.0967	17.5240	18.6358	16.9788	19.8553	0.079
**20:1**	Eicosenoic	14.2859	12.5524	20.9498	21.0880	15.3532	25.7045	0.125	0.1278	0.0963	0.1452	0.1162	0.1074	0.1251	0.335
**22:1**	Erucic (n-9)	10.0617	9.3359	10.5904	11.9576	10.7772	14.0745	0.014*	0.0756	0.0570	0.0923	0.0650	0.0590	0.0769	0.289
**24:1**	Nervonic (n-9)	46.8990	41.2338	52.4328	54.9080	49.9858	65.7098	0.064	0.3622	0.2600	0.5070	0.3225	0.2536	0.3583	0.304
**26:1**	Hexacosaenoic	0.9553	0.8910	1.1118	1.3843	1.0353	1.7047	0.013*	0.0075	0.0057	0.0089	0.0070	0.0051	0.0080	0.728
**18:2**	Linoleic (n-6)	2791.7802	2070.6058	3453.9837	3792.3849	2929.4188	4962.5098	0.022*	20.6984	19.9908	21.1132	21.1801	20.4337	22.6097	0.110
**20:2**	Eicosadienoic	18.3719	14.3488	22.0766	23.7914	18.2850	32.1048	0.125	0.1420	0.1162	0.1666	0.1342	0.1188	0.1509	0.728
**22:2**	Docosadienoic	1.1531	0.8263	1.3466	1.0588	0.8793	1.3235	0.912	0.0071	0.0059	0.0086	0.0053	0.0043	0.0073	0.034
**18:3**	Octadecatrienoic (linolenic)	140.7094	103.1703	211.9496	167.0206	135.9088	362.9094	0.200	1.0063	0.8499	1.5595	1.0568	0.7545	1.5950	0.837
**20:3**	Eicosatrienoic/Dihomo-γ-linolenic	174.4106	123.2436	208.0762	232.2868	205.0980	275.8734	0.016*	1.1988	1.0496	1.3663	1.2025	1.0405	1.3291	0.887
**18:4**	Stearidonic	2.0618	1.8962	3.4901	2.1916	1.5408	3.2393	0.937	0.0167	0.0095	0.0235	0.0122	0.0090	0.0156	0.129
**20:4**	Eicosatetraenoic/Arachidonic	2151.3579	1622.8461	2742.0318	2924.8647	2280.8060	3542.4020	0.016*	15.9281	13.1892	17.1341	17.2232	14.9086	18.3354	0.319
**22:4**	Docosatetraenoic (Adrenic)	52.0539	28.8466	66.3910	53.4361	40.3768	81.8793	0.289	0.3366	0.2232	0.4217	0.3543	0.1959	0.4361	0.937
**20:5**	Eicosapentaenoic	208.5916	131.8676	411.7202	174.9416	66.1434	259.7677	0.438	1.4249	0.7772	3.7935	0.9776	0.5452	1.4203	0.103
**22:5**	Docosapentaenoic	217.8699	182.8768	249.3548	211.6448	164.8822	280.8601	1.000	1.4465	1.2051	1.7658	1.1522	0.9180	1.4818	0.056
**22:6**	Docosahexaenoic	261.9314	146.2513	369.6664	159.4761	85.1149	329.5998	0.189	2.1143	1.1406	3.0117	0.8629	0.5299	1.4849	0.012*

*Retained significance at a Benjamini-Hochberg corrected p-value <0.15.

### Complex lipids

**Fig 5:**
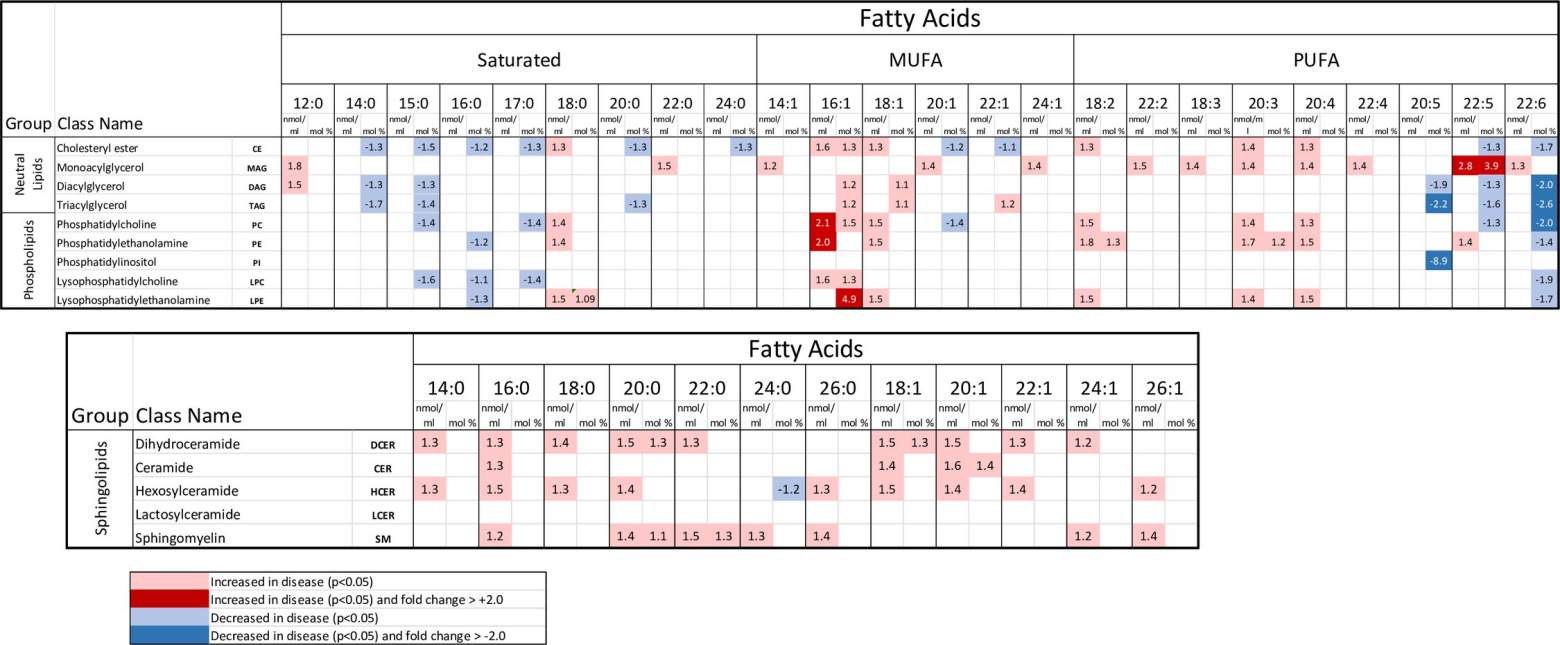
Classes and associated fatty acid composition of complex lipids whose concentrations and mol % composition were measured as significantly different (uncorrected p-value <0.05) in plasma from 18 dogs with gallbladder mucocele formation and 18 dogs matched by age and breed. For each lipid species, numbers reported represent fold change between gallbladder mucocele and control and are shown for both concentration (nmol/ml) and composition (mol %). Blue shaded values represent significant decrease with dark blue representing fold change > -2.0. Pink shaded values represent a significant increase with red representing fold change > 2.0.

**Fig 6:**
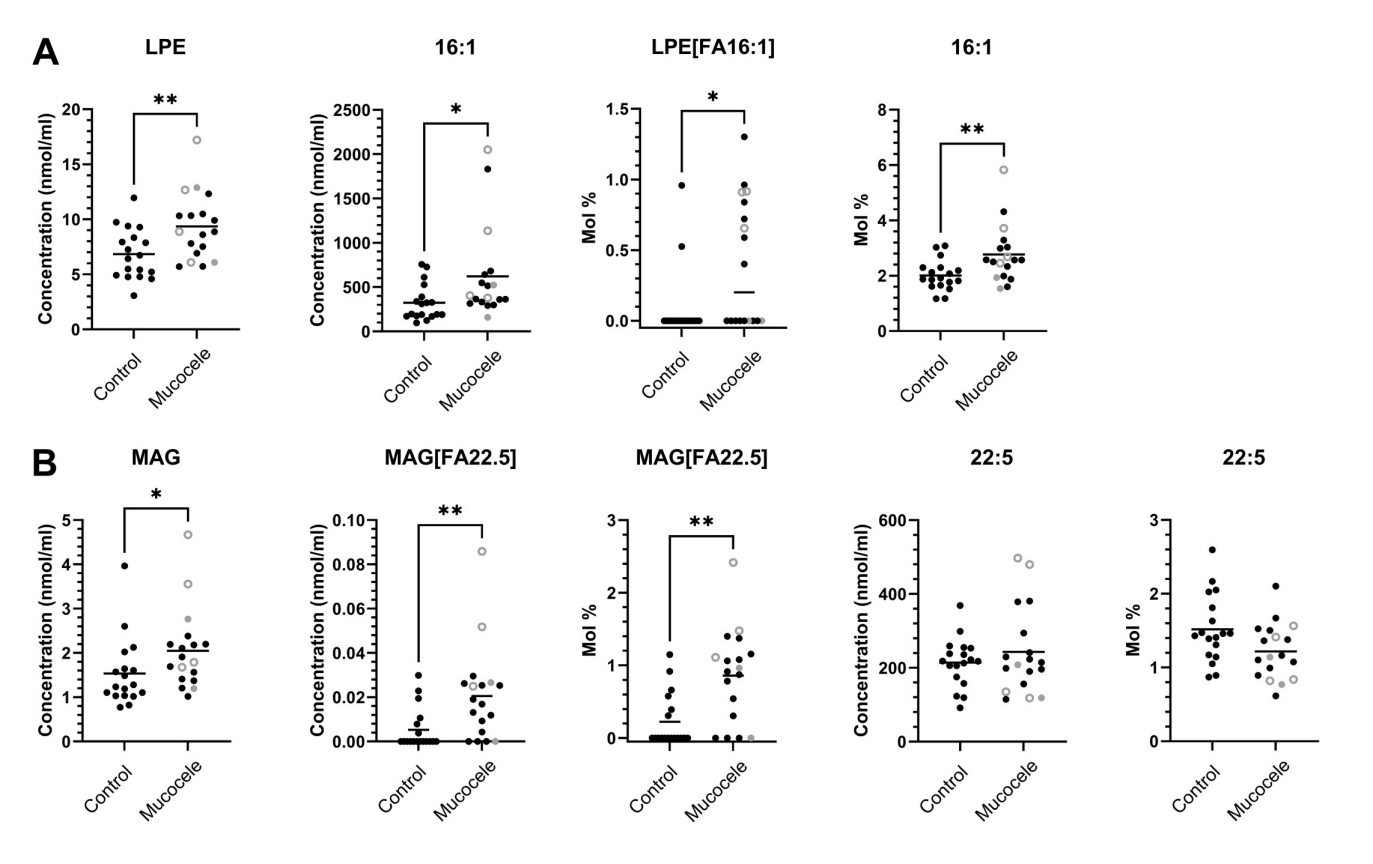
Complex lipid species and associated esterified fatty acids identified as significantly greater in concentration (nmol/ml) or relative abundance (mol %) in plasma from 18 dogs with gallbladder mucocele formation compared to 18 dogs matched by age and breed. Open circles represent dogs having serum biochemical evidence of cholestasis as defined by a serum total bilirubin concentration greater than the upper end of the reference range (>0.2 mg/dl). Gray datapoints represent dogs with illness severity score ≥ 2. Mann-Whitney P value *<0.05, **<0.01.

Among the 981 unique complexes of class and esterified fatty acid(s) lipid species measured, 10.8% were significantly increased while 0.0% were significantly decreased in plasma concentration comparing dogs with gallbladder mucocele formation to control dogs. Increased complex lipid concentrations largely mirrored the documented **(Table 3**) increases in associated individual fatty acid concentrations, particularly regarding palmitoleic (16:1) (**Fig 5**, nmol/ml). On a mol % basis the relative abundance of some specific fatty acid esterifications were significantly over or under-represented within certain classes (**Fig 5**, mol %). Significant increases in relative abundance were notable for palmitoleic (16:1) across multiple classes and was of greatest magnitude for lysophosphatidylethanolamine in which palmitoleic was 4.9 fold increased compared to other fatty acids (**Fig 6**). An additional increase in both concentration and relative abundance was notable for esterification of monoacylglycerol with docosapentaenoic (22:5) wherein 22:5 had a 3.9 fold increase compared to other fatty acids (**Fig 6**). The majority of significant changes in relative abundance of fatty acids within specific lipid classes demonstrated decreases and included those same fatty acids previously identified as present at a significantly lower relative abundance compared to other fatty acids (**Table 3**), namely pentadecanoic (15:0), margaric (17:0), and docosahexaenoic (22:6). Decreases were notable in magnitude for the omega-3 fatty acids docosahexaenoic (22:6) and eicosapentaenoic (20:5) across several classes but largest for phosphatidylinositol in which eicosapentaenoic (20:5) was -8.88 fold decreased compared to other fatty acids (**Fig 5**, mol %).

### Cytochemistry and transmission electron microscopy

**Fig 7:**
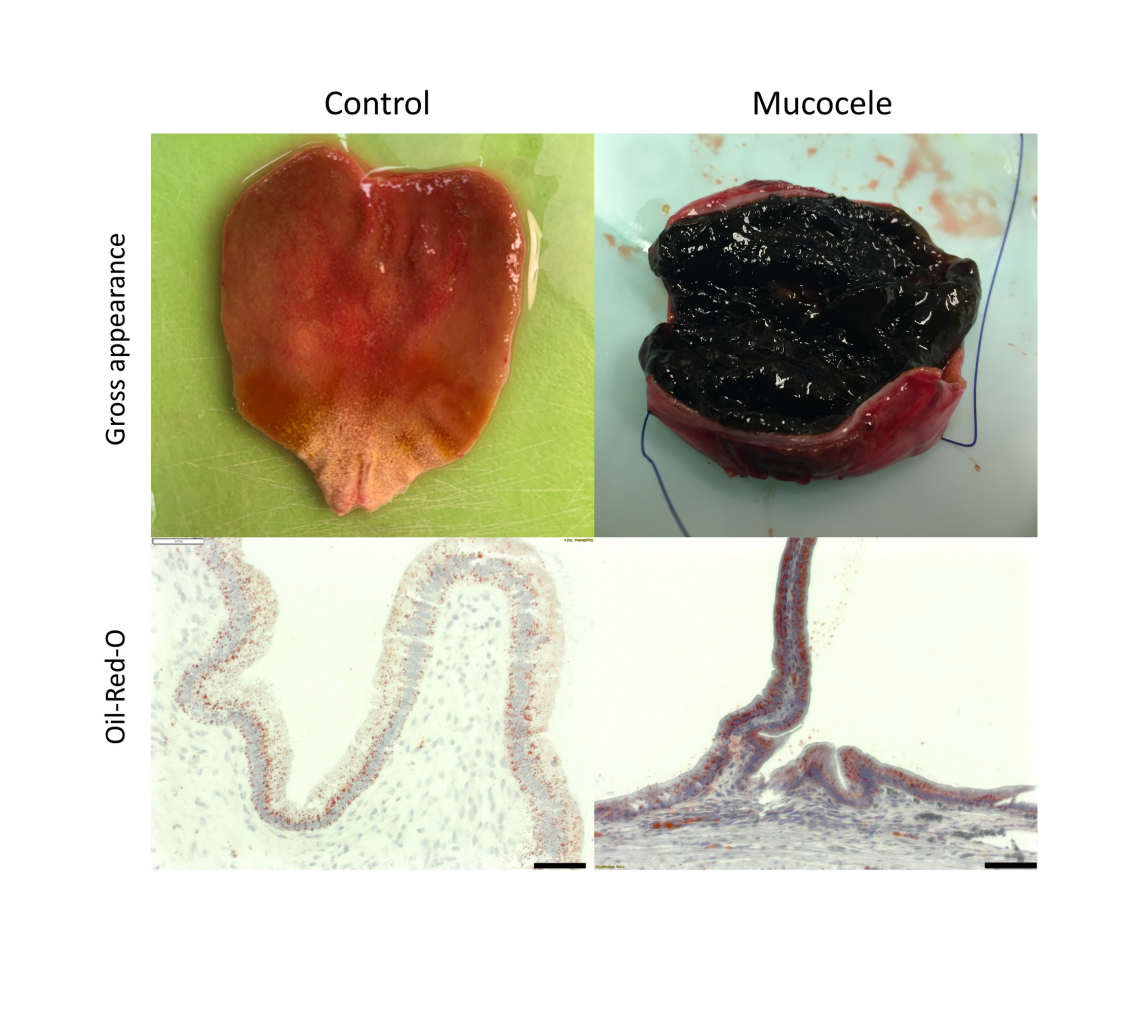
Representative gross and light microscopic appearance of gallbladder epithelium from a control (left panels) and mucocele (right panels) gallbladder after staining with the neutral fat lipophilic stain Oil-Red-O.

**Fig 8:**
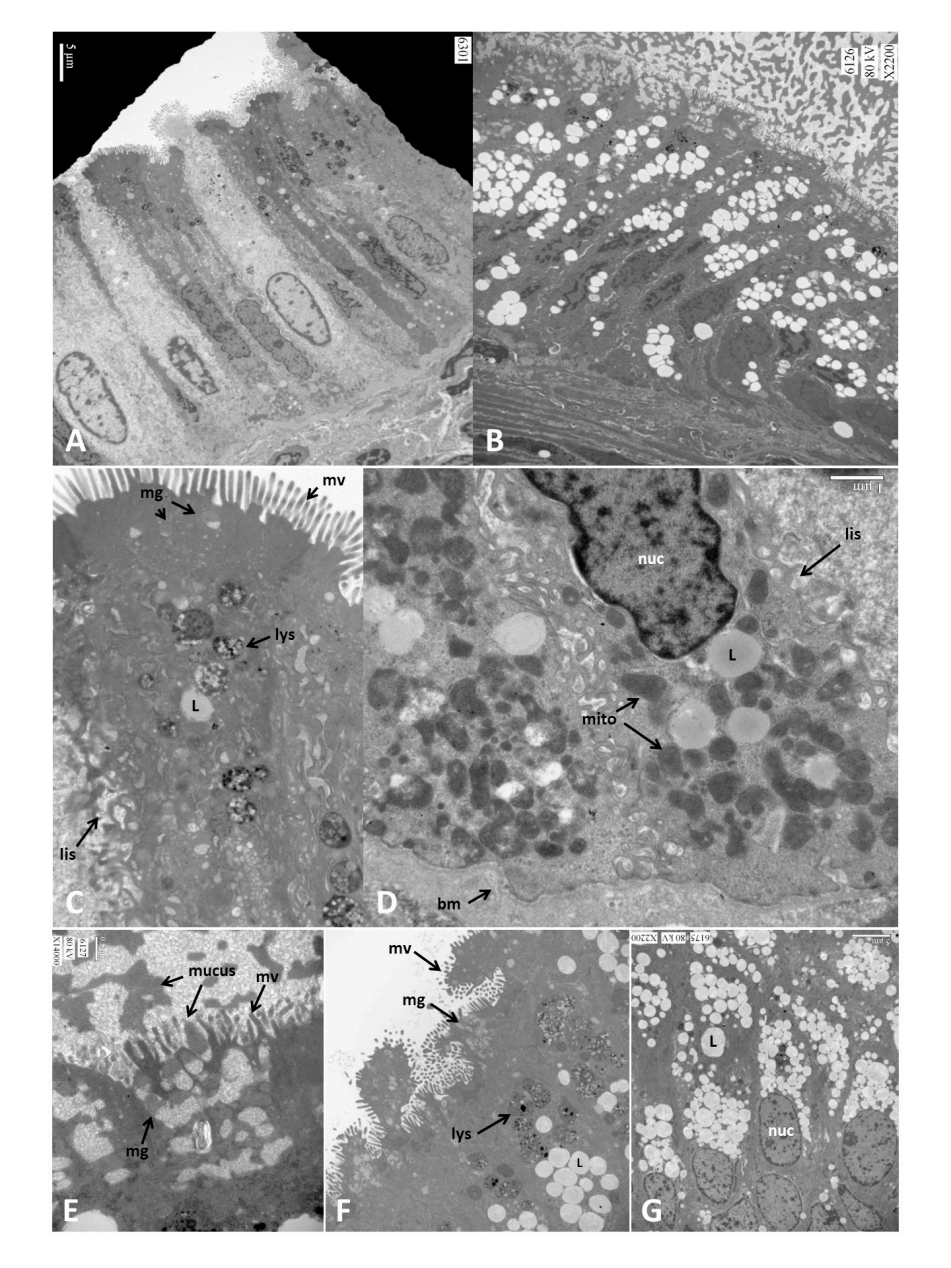
Transmission electron microscopic images of control (panel A, C, and D) and mucocele gallbladder epithelium (panel B, E, F, and G). mv; microvilli, mg; mucus granule, lys; lysosome, L; lipid droplet, lis; lateral intercellular space, nuc; nucleus, bm; basement membrane, mito; mitchondria.

Mucosa was collected from 2 control and 4 mucocele canine gallbladders and underwent examination of the epithelium after staining for neutral fats using Oil-Red-O and via transmission electron microscopy. Light microscopic examination of frozen sections after staining with Oil-Red-O confirmed an increased accumulation of neutral lipid in mucocele gallbladder epithelium (**Fig 7**). Visualized by transmission electron microscopy, control gallbladder epithelium consisted of tall columnar cells varying in cytoplasmic electron density between “light” and “dark” cells as previously described [18] (**Fig 8A**). Epithelial cells had apical membrane microvilli, an interdigitating membrane along the lateral intercellular space, a basement membrane, and basally located nuclei. The cytoplasm residing beneath the apical membrane contained small numbers of mucus granules containing amorphous mucus, larger electron dense lysosomes, and infrequent lipid droplets. The subnuclear cytoplasm contained numerous round to oval organelles interpreted to be mitochondria (**Fig 8A, 8C, and 8D**). Epithelial cells lining mucocele gallbladders were remarkable for their content of mucus granules and lipid droplets (**Fig 8B**). Large projections into the lumen of microvilli-lined apical membrane containing apical cytoplasm and mucus granules were observed in some sections (**Fig 8F**). The apical cytoplasm was filled with copious mucus granules containing dense mucus and frequently seen in the process of exocytosis into the lumen (**Fig 8E**). Subtending the mucus granules were lysosomes underlaid by copious numbers of variably sized lipid droplets throughout the supranuclear and subnuclear cytoplasm (**Fig 8G**).

## Discussion

The main objective of this study was to increase understanding of the characteristics and pathogenesis of hyperlipidemia which is a common comorbidity in dogs diagnosed with gallbladder mucocele formation. Hyperlipidemia in dogs with gallbladder mucocele formation has not been previously characterized beyond identification of hypercholesterolemia (41–55% of dogs), hypertriglyceridemia (43–54% of dogs), or both (~14% of dogs) in serum samples obtained for diagnostic purposes [2, 8]. The relationship between gallbladder mucocele formation and hyperlipidemia is further confounded by predisposition for mucocele formation among several breeds with known risk for hypercholesterolemia or hypertriglyceridemia (e.g. Shetland sheepdog and miniature schnauzer). The present study attempted to control this variable using a breed-matched cohort of control dogs but nonetheless cannot eliminate the possibility that some breeds with a mucocele may have been more likely to have or develop a lipidemic disorder. Sixty-one percent of dogs in this study had hypercholesterolemia. Serum based enzymatic assay of triglycerides was not concurrently performed in these dogs and therefore prevalence of hypertriglyceridemia cannot be directly compared to prior clinical reports. Using mass spectrometry, we did not identify significant differences in median plasma triacylglyceride concentrations between control dogs and dogs with gallbladder mucocele formation.

Using an infusion mass spectrometry-based lipidomics approach, our results document that 50% of lipid classes, 36% of esterified fatty acid species, and 11% of complex lipid species identified were significantly increased in plasma concentration in dogs with gallbladder mucocele formation. Given that each dog was fasted and concurrent endocrinopathy was established as unlikely by diagnostic testing, these findings are likely to reflect an endogenous increase in lipogenesis. In addition, features of dyslipidemia in this study, as well as a prior global metabolomics study of dogs with gallbladder mucocele formation [11], could not be simply attributed to cholestasis (concurrent hyperbilirubinemia) or severity of illness in individual dogs.

A remarkable finding in this study was a significant increase in concentration and relative abundance of palmitoleic acid (16:1) across numerous classes of neutral lipids, phospholipids, and lysophospholipids in dogs with gallbladder mucocele formation. Dietary sources of naturally occurring palmitoleic acid are limited and most is produced endogenously by the actions of stearoyl-CoA desaturase 1 (SCD-1) giving rise to the *cis* isoform, *cis*-palmitoleate (n-7; Δ9). In people, *cis*-palmitoleate biosynthesis occurs principally in the liver, and secondarily in adipose tissue, where it is incorporated into triglycerides, phospholipids, and cholesterol esters. A significant increase in the ratio of palmitoleic to palmitic acid (16:1/16:0) in dogs with gallbladder mucocele formation across multiple lipid classes indirectly supports an increase in SCD-1 activity [19]. In people, both SCD-1 activity and plasma palmitoleic acid concentration are stimulated by carbohydrate excess and serve as key drivers for de novo lipogenesis and the hyperlipidemia associated with metabolic syndrome [20, 21]. It is worth acknowledging that consumption of whole fat dairy products can provide a minor exogenous source of palmitoleic acid in the form of a *trans* isomer [22, 23]. While diet history was not exhaustive for dogs included in the current study, dogs that formed a gallbladder mucocele did not appear to have a unique diet history that could explain an increased intake of palmitoleic acid. In fact, decreases in the odd chain fatty acids 15:0 and 17:0, whose primary source is dairy fat, do not support an increase in milk fat intake. Nonetheless, studies to distinguish *cis-* from *trans-*palmitoleic acid have a potential to further clarify the origin and physiological impact of palmitoleic acid in dogs with gallbladder mucocele formation as these isomers have been associated with unreconciled differential influences on chronic metabolic diseases such as obesity, hepatosteatosis, insulin sensitivity, and cardiovascular disease (reviewed in [24]). It is of interest that results of our prior untargeted global mass spectrometry-based analysis of serum samples from dogs with gallbladder mucocele formation also supported systemic changes consistent with energy excess and metabolic syndrome. For example, hypercholesterolemia in that study was associated with concurrent increases in lanthosterol and 7α-hydroxycholesterol supporting a primary increase in cholesterol and bile acid synthesis [11]. What remains unclear is the driving force for lipogenesis in these dogs.

Another significant finding in this study was a decrease in abundance of the esterified omega-3 fatty acids, eicosapentaenoic (EPA 20:5), docosapentaenoic (DPA 22:5), and docosahexaenoic (DHA 22:6) across multiple classes of complex lipids in dogs with gallbladder mucocele formation. Using global untargeted mass spectrometry we previously demonstrated significant increases in the free fatty acid counterparts to these omega-3 fatty acids in serum and bile from dogs with gallbladder mucocele formation [11]. A direct comparison between this study and our prior metabolomics study is somewhat problematic as the untargeted study was semiquantitative, not case-controlled, and performed on dogs in which concurrent influence of endocrinopathy was not ascertained. In this study, an exception to the relative decrease in abundance of esterified omega-3 fatty acids was an isolated increase in concentration and mol % of 22:5 associated with monoacylglycerol (MAG [22:5]). A major limitation of our approach here was reliance on differential mobility spectrometry that involves no chromatography steps to separate positional isomers. Accordingly, we cannot resolve fatty acid 22:5 ω-3 from its lower abundance isomer 22:5 ω-6 which limits our interpretation of this finding. Finally, whether these changes represent an overall imbalance in the ratio of omega-3 to omega-6 fatty acids remains an important question and potential opportunity for nutritional intervention. For example, studies in cholesterol-fed prairie dogs have shown that dietary supplementation with omega-3 fatty acids can decrease the arachidonic acid (omega-6) content of phospholipids in gallbladder mucosa and bile resulting in decreased prostaglandin synthesis [25]. In gallbladder epithelial cells from dogs, prostaglandins can stimulate mucin secretion [26, 27].

An enigmatical observation in dogs with mucocele formation is the striking severity of disease involving the gallbladder but also a systemic disturbance in lipid metabolism and endocrinopathy. In the present study we identified additional ultrastructural evidence for abnormal lipid metabolism and/or transport in gallbladder epithelium of dogs with mucocele formation. Whether this lipidosis is a consequence of or related to the cause of hyperlipidemia or gallbladder mucocele formation in these dogs is unclear. Future studies designed to examine changes in the plasma lipidome and gallbladder lipid over the course of mucocele development may shed further light on this relationship. Prior descriptions of the ultrastructural appearance of canine gallbladder epithelium are infrequent [18, 28–30] but include a toxicology study reporting gallbladder epithelial lipidosis in dogs treated with an inhibitor of leukotriene biosynthesis. Unique to the dogs in that report was an infiltration of the lamina propria with macrophages containing lipid and cholesterol clefts, similar to descriptions in people with gallbladder cholesterolosis [31]. Cholesterolosis lesions were not observed in gallbladder mucocele tissues in this study. There exists a short case report of gallbladder pathology strikingly similar to gallbladder mucocele formation in a dog treated with progestational compounds in which increased amounts of neutral fat was demonstrated in epithelial cells [32]. In an additional toxicological study, dogs fed cholesterol and treated with propylthiouracil (to inhibit thyroid hormone synthesis) developed lipidosis restricted to the epithelium of the gallbladder and intrahepatic bile ducts that corresponded with accumulated cholesterol and cholesterol esters presumably absorbed from the bile [33]. The gallbladder normally absorbs biliary cholesterol and phosphatidylcholine in proportion to their concentration in bile to prevent cholesterol precipitation [34]. The idea that lipidosis of gallbladder epithelium is somehow related to taxation of cholesterol transport is compelling, particularly in light of the association of gallbladder mucocele formation with hypercholesterolemia as well as hypothyroidism. Abnormal gallbladder cholesterol transport may also explain the pathogenesis of atherosclerotic lesions described in the gallbladder of a dog with mucocele formation [35] and dogs with gallbladder vascular infarction [36].

Results obtained from the dogs included in this study support a primary increase in lipogenesis in association with gallbladder mucocele formation and lipidosis of gallbladder epithelium with features reminiscent of abnormal cholesterol metabolism. Further insight into strategies for nutritional or pharmacological intervention may be gained by quantitative analysis of free fatty acids including separation of cis and trans fatty acids and quantification of different positional isomers. Finally, the studies identify a compelling rationale for further characterization of gene expression and signaling pathways regulating abnormal lipid metabolism in the liver and gallbladder of dogs with mucocele formation.

## Supporting information

S1 Fig Plasma triacylglyceride concentration in dogs with gallbladder mucocele formation compared to age and breed matched control dogs. Open circles represent dogs having serum biochemical evidence of cholestasis as defined by a serum total bilirubin concentration greater than the upper end of the reference range (>0.2 mg/dl). Gray datapoints represent dogs with illness severity score ≥ 2. Mann-Whitney test.
(TIF)

S1 Table Diet, oral medication, and supplement history reported in medical records of dogs in this study. (DOCX)

S2 Table Excel file of raw lipidomics data. (XLSX)
