# Supplementary figures and images for: Increased lipogenesis and lipidosis of gallbladder epithelium in dogs with gallbladder mucocele formation
Source: PLoS One. 2024 Jun 26;19(6):e0303191. doi: 10.1371/journal.pone.0303191 (PMC11207163; doi:10.1371/journal.pone.0303191)

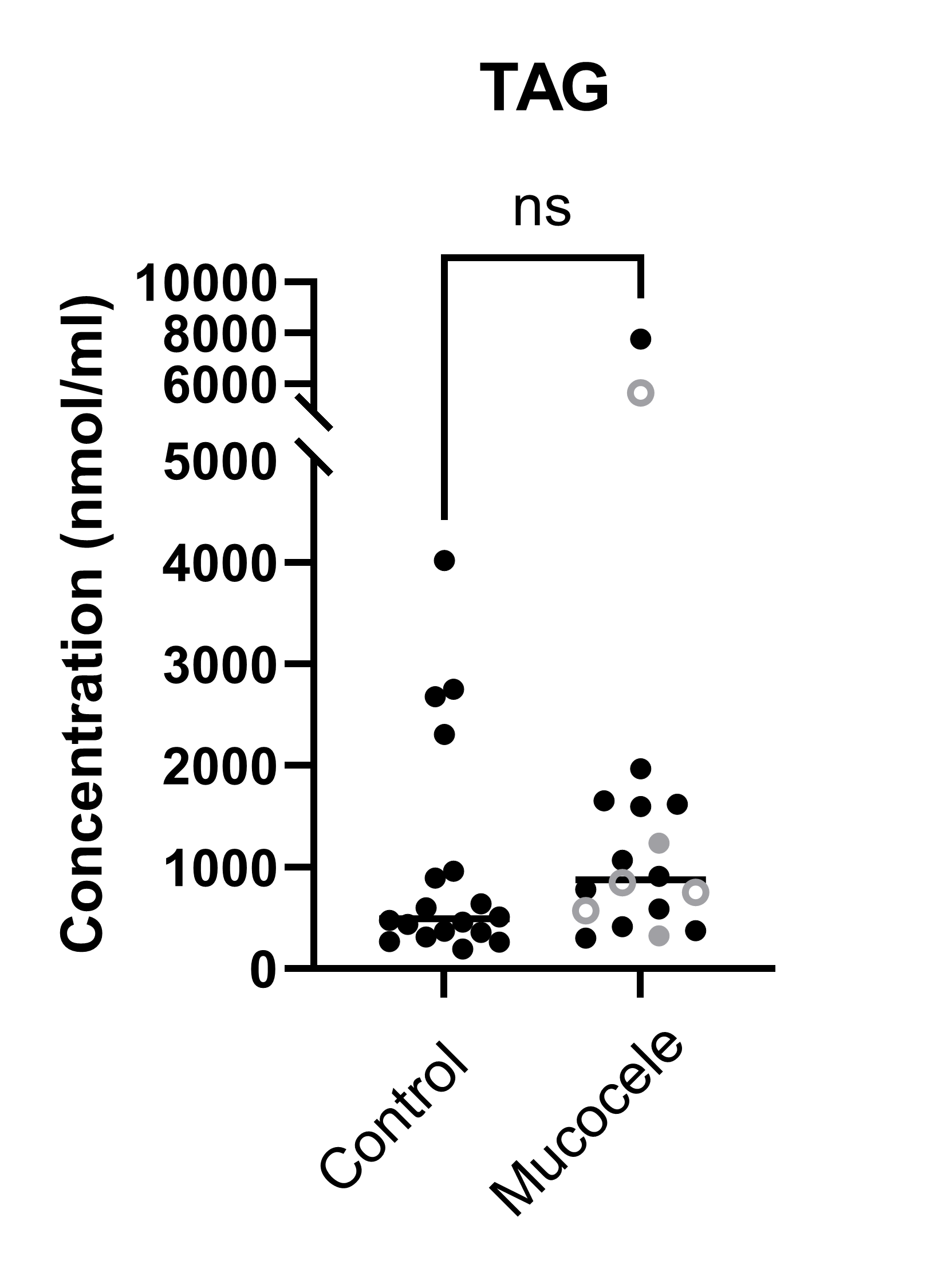

Supplement: S1 Fig — Open circles represent dogs having serum biochemical evidence of cholestasis as defined by a serum total bilirubin concentration greater than the upper end of the reference range (>0.2 mg/dl). Gray datapoints represent dogs with illness severity score ≥ 2. Mann-Whitney test. (TIF) [file pone.0303191.s001.tif]
