# Supplementary material for: Increased lipogenesis and lipidosis of gallbladder epithelium in dogs with gallbladder mucocele formation
Source: PLoS One. 2024 Jun 26;19(6):e0303191. doi: 10.1371/journal.pone.0303191 (PMC11207163; doi:10.1371/journal.pone.0303191)
Supplement: S1 Table — (DOCX) [file pone.0303191.s002.docx]

**S1 Table.**  Diet, oral medication, and supplement history reported in medical records of dogs in this study.

| **Diet and medication** | **Control dogs (n=18)** | **Mucocele dogs (n=18)** |
| --- | --- | --- |
|  | **No (%)** | **No (%)** |
| **Any diet history available** | 12 (67) | 14 (78) |
| Purina | 5 (42) | 1 (7) |
| Hills | 1 | 3 |
| Blue buffalo | 0 | 2 |
| Wellness | 0 | 2 |
| Royal canin | 1 | 1 |
| Fromms | 1 | 1 |
| Earthborne | 1 | 0 |
| Pedigree | 1 | 0 |
| Lotus | 1 | 0 |
| Kirkland | 1 | 0 |
| Iams | 1 | 0 |
| Pet pantry | 0 | 1 |
| Natures domain | 0 | 1 |
| Taste of the wild | 0 | 1 |
| Natural balance | 0 | 1 |
| **Any main ingredient info available** | 8 (44) | 11 (61) |
| Fish (Herring meal, Salmon, Mackeral,  ocean whitefish) | 5 (62.5) | 5 (45) |
| Chicken | 4 (50) | 5 (45) |
| Lamb | 2 | 0 |
| Rabbit | 1 | 0 |
| Duck | 0 | 2 |
| Beef | 0 | 1 |
| **Any flea, tick, or heartworm preventative** | 12 (67) | 16 (89) |
| Ivermectin | 8 (67) | 6 (37.5) |
| Milbemycin | 4 (33) | 7 (44) |
| Fipronil | 2 (17) | 4 (25) |
| Imidacloprid | 5 (42) | 0 (0) |
| Afoxolaner | 4 (33) | 0 (0) |
| Lufenuron | 0 (0) | 4 (25) |
| Spinosad | 2 | 1 |
| Moxidectin | 1 | 0 |
| Flurilaner | 1 | 0 |
| Flumethrin | 1 | 0 |
| Salamectin | 0 | 1 |
| Etofenprox | 0 | 1 |
| Indoxacarb | 0 | 1 |
| **Any oral medications and supplements** | 3 (17) | 11 (61) |
| Cranberry extract | 2 | 0 |
| Amlodipine | 0 | 1 |
| Amoxicillin | 0 | 1 |
| B-complex vitamins | 0 | 1 |
| Carprofen | 0 | 1 |
| Denamarin | 0 | 1 |
| Diethylstilbesterol | 1 | 0 |
| Doxycycline | 0 | 1 |
| Enalapril | 1 | 3 |
| Enrofloxacin | 0 | 1 |
| Famotidine | 0 | 2 |
| Finasteride | 0 | 1 |
| Gabapentin | 1 | 0 |
| Insulin | 0 | 1 |
| Joint supplement | 0 | 2 |
| Maropitant | 1 | 1 |
| Metaclopramide | 0 | 1 |
| Metamucil | 0 | 1 |
| Methocarbamol | 1 | 0 |
| Metronidazole | 1 | 1 |
| Phenoxybenzamine | 0 | 0 |
| Phenylpropanolamine | 1 | 0 |
| Pimobendan | 0 | 1 |
| Theophylline | 0 | 1 |
| Wellactin | 1 | 0 |
| Zinc | 0 | 1 |
